# Influence of Concentration and Activation on Hydrogen Peroxide Diffusion through Dental Tissues *In Vitro*

**DOI:** 10.1155/2013/193241

**Published:** 2013-09-18

**Authors:** Carlos R. G. Torres, Cristiane S. Souza, Alessandra B. Borges, Maria Filomena R. L. Huhtala, Taciana M. F. Caneppele

**Affiliations:** Department of Restorative Dentistry, Institute of Science and Technology, Universidade Estadual Paulista (UNESP), Avenue Francisco José Longo 777—12245-000 São José dos Campos, SP, Brazil

## Abstract

This study evaluated the effect of physical and chemical activation on the diffusion time of different concentrations of hydrogen peroxide (HP) bleaching agents through enamel and dentin. One hundred and twenty bovine cylindrical specimens were divided into six groups (*n* = 20): 20% HP; 20% HP with light activation; 20% HP with manganese gluconate; 35% HP; 35% HP with light activation; and 35% HP with manganese gluconate. The specimens were fixed over transparent epoxy wells with internal cavities to simulate a pulpal chamber. This chamber was filled with an enzymatic reagent to simulate pulpal fluid. The bleaching gels were applied on enamel surface and the image of the pulpal fluid was captured by a video camera to monitor the time of peroxide penetration in each specimen. ANOVA analysis showed that concentration and type of activation of bleaching gel significantly influenced the diffusion time of HP (*P* < 0.05). 35% HP showed the lowest diffusion times compared to the groups with 20% HP gel. The light activation of HP decreased significantly the diffusion time compared to chemical activation. The highest diffusion time was obtained with 20% HP chemically activated. The diffusion time of HP was dependent on activation and concentration of HP. The higher concentration of HP diffused through dental tissues more quickly.

## 1. Introduction

Dental bleaching is a noninvasive treatment that can result in satisfactory esthetic outcomes. It is based on the ability of hydrogen peroxide to penetrate through tooth structure and produce free radicals that oxidize the colored organic molecules [1].

The different hydrogen peroxide concentrations used for bleaching procedures lead to a greater or lesser effect on the pulpal tissue, since there is evidence of penetration of the bleaching agents through the tooth structure and, hence, tissue irritation, which may cause tooth sensitivity during the bleaching [2]. This sensitivity may be a result of the diffusion of hydrogen peroxide or other toxic components along with the degradation of the bleaching gel [3, 4].

In order to accelerate the releasing of free radicals, professionals have used devices that transfer energy to hydrogen peroxide, increasing its decomposition. The activating sources are not responsible for the bleaching process properly; they only intend to increase the degradation of the bleaching gel, that is, the one actually responsible for the bleaching effect. In general, the activating sources used are the halogen lamp, light emitting diode (LED), plasma arc, and laser [5]. However, light application may cause a temperature rise [6] and increase the diffusion of the bleaching agent through the dental hard tissues that reaches the pulpal chamber. The hydrogen peroxide diffusion through enamel and dentin is facilitated by its low molecular weight and capacity to denature proteins [7]. Previous studies have demonstrated cytotoxic effects of 35% hydrogen peroxide bleaching gel on pulpal cells [8, 9].

As an attempt to increase the efficacy and maintain the safety of the treatment, chemical activating agents have been incorporated in the bleaching gels [10–12]. Substances derived from metals such as manganese and iron or enzymes such as catalase and peroxidase can react with hydrogen peroxide, accelerating the production of free radicals. This aims to obtain a faster, more efficient, and safer bleaching process and to reduce the potential damaging effects associated with the application of heat sources [12]. In addition, there may be some influence on the penetration of hydrogen peroxide, since a reaction is expected to occur between the hydrogen peroxide and the chemical activator [10]. 

Another alternative to decrease the pulpal damage is the use of lower-concentrated hydrogen peroxide gels in the in-office bleaching. However, there is no consensus about its efficacy and influence on pulpal penetration. Pulpal damage has been and is still a concern regarding bleaching treatments.

Since different concentrations of hydrogen peroxide and methods of activation can influence the diffusion of the product through dental tissues, the aim of this study was to evaluate the effect of concentration and activation of hydrogen peroxide gels on the diffusion time through enamel and dentin. It was hypothesized that (1) the higher concentration of the hydrogen peroxide gel and (2) the activation mode (light or chemical) would affect the diffusion time through enamel and dentin.

## 2. Material and Methods

One hundred and twenty extracted, nondamaged, and intact bovine incisors were stored in 0.1% thymol solution at room temperature. From each crown, one specimen 6 mm in diameter and 2 mm in height (1 mm of enamel and 1 mm of dentin) was prepared from the buccal surface with the aid of a trephine mill.

The enamel-dentin (ED) disks were randomly distributed into two groups: 20% hydrogen peroxide and 35% hydrogen peroxide bleaching gels. Each group was divided into three subgroups: light activated, chemically activated, and no activation. For all groups, 3 × 10 min applications of hydrogen peroxide gel were performed.

The light activation was performed using a blue LED device (Bright Max II, MM Optics Ltda, São Carlos, SP, Brazil) three times, 2 min each, with an interval of 1 min, and the chemical activation was performed by adding 0.025% of manganese gluconate ([CH_2_OH(CHOH)_4_COO]_2_Mn·2H_2_O; Gluconal-Purac, Campo de Goytacazes, RJ, Brazil).

Transparent epoxy wells (Arotec, São Paulo, SP, Brazil), with an internal cavity capacity of 20 *μ*L, were used to simulate a pulpal chamber. The chamber was filled with 10 *μ*L of an enzymatic reagent, proposed by Bauminger [13] and modified by Hannig et al. [14–16], used to simulate the pulpal fluid and reveal the presence of hydrogen peroxide. It is based on the reaction of 4-aminoantipyrin and phenol with hydrogen peroxide catalyzed by peroxidase. The inorganic peroxide is oxidized by peroxidase, releasing oxygen which oxidizes achromatic chromogenic hydrogen donors, changing the color of the solution from transparent to pink. After placement of the reagent, the specimens were positioned into the wells, with the dentin side in contact with the enzymatic solution, and an O-ring was placed over them. A cap was threaded to ensure a perfect sealing. The quality of the seal provided by the O-rings was tested using specimens with dimensions identical to the dental structure but in resin, thus, eliminating the dental permeability. In this test, no penetration of hydrogen peroxide was detected inside the chambers, demonstrating the adequate sealing.

The bleaching treatment was performed according to each activating mode, using an experimental 20% or 35% hydrogen peroxide bleaching gel. This gel is composed of two parts. One part, Solution A (pH 1.5), is a solution of hydrogen peroxide containing an acrylic thickener, which in an acidic environment is a white solution. The other part, Solution B (pH 11.3), consists of an aqueous solution containing an alkaline substance, in which the chemical activator was added or not. An orange dye was also added to Solution B to absorb the blue light during light activation of the gel. To obtain the final bleaching gel (pH 6.5), three parts of Solution A and one part of Solution B, in volume, were mixed in a mixing well. To be certain of the hydrogen peroxide concentration of the hydrogen peroxide solution (Synth, Diadema, SP, Brazil) used to prepare the gel, the titration method using 0.1 N potassium permanganate (KMnO_4_) solution was used. Primary standard sodium oxalate was used to standardize the 0.1 N KMnO_4_ [17]. Bleaching was performed in a humid atmosphere. The metal salt used as the chemical activator is not toxic when used in the right concentration, since it is used as a nutritional supplement in human diet.

The bleaching agents (20 *μ*L) were applied on the enamel surface of the specimens and kept in place for 10 min. The gel was aspirated and the sample surface was washed with air spray/deionized water. The surface was dried with paper towels, and artificial saliva was applied to keep the enamel hydrated. This procedure was repeated three times.

**Figure 1:**
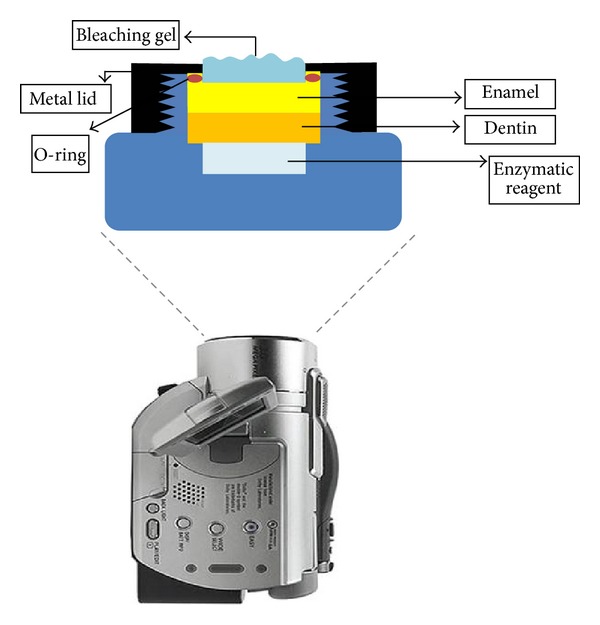
Schematic drawing of the sample holder.

A digital camera (Sony DCR-DVD 405, Sony Corporation, Japan) was fixed laterally to the sample holders, in order to film the chamber and record the time required for the enzyme reagent to change color (Figure 1). The timer counting was initiated with the application of the bleaching gel.

The data were analyzed with two-way ANOVA and Tukey's tests. All the analyzes were conducted using the software Statistica for Windows (StatSoft, Tulsa, Oklahoma, USA) with a level of significance at 5%. The factors analyzed were the concentration of hydrogen peroxide (20 and 35%) and the mode of activation (light, chemical, or no activation).

## 3. Results

The overall time necessary for hydrogen peroxide to reach the pulp chamber for all experimental conditions is shown in Table 1. The cross-product concentrations versus the activation as well as the concentration and activation factors were statistically significant (*P* < 0.02).

For 35% hydrogen peroxide, no significant differences were observed for the activation modes in comparison to control group (no activation). However, for 20% hydrogen peroxide, significant differences were observed between the light activated and the chemically activated groups.

Table 2 shows the results of the concentration of hydrogen peroxide as a factor. The average time needed for the hydrogen peroxide to reach the simulated pulp chamber is significantly shorter for the higher concentrated bleaching gel.

Table 3 shows the results of Tukey's test for the method of activation as a factor, and a significant difference was observed only between chemical and light activation.

## 4. Discussion

Both hypotheses of this study were accepted, since the concentration and the mode of activation of bleaching gels have affected the diffusion time through enamel and dentin.

The 35% HP diffused quicker than 20% HP through enamel and dentin. A recent study [18] investigated the diffusion time necessary for the hydrogen peroxide bleaching gel through enamel and dentin by micro-Raman spectroscopy. The authors observed that HP penetrated enamel, reaching the underlying dentin, oxidizing its organic compounds, and modifying its mineral compounds. The marked increase of the Raman band near dentin enamel junction is due the fact that this is the most organic region of the dentin layer, thus, showing the affinity of HP for organic compounds.

It might be speculated that the renewal of the pulp fluids could decrease the toxicity of hydrogen peroxide when reaching the pulp chamber. Thus, the longer the diffusion of peroxide takes to reach the pulpal chamber the less toxic is the product, because there is more time to dilute and degrade the peroxide that reaches the pulp. Therefore, since some studies showed no significant differences regarding color changes when comparing gels of lower concentrations with higher concentrations [19, 20], the lower-concentrated agents may be less detrimental to pulpal tissue, without impairing efficacy.

In this study, the light activation decreased the diffusion time for the 20% hydrogen peroxide gel but not for the 35%. Light activation leads to gel heating. As temperature rises, molecules move faster, and the whole system acquires more kinetic energy. According to the molecular collisions theory, during a collision, part of the kinetic energy is used, so that bonds are weakened and broken, making the reaction possible. So, with the temperature rise, the collisions among the gel molecules turn to be more frequent, increasing the likelihood of bonding cleavages and reactions and accelerating the diffusion speed [21].

The results of *in vivo* studies that compared vital tooth bleaching therapies with and without light activation are controversial regarding the real contribution of light irradiation to the final outcome of dental bleaching therapies [22–24]. Dias Ribeiro et al. [9] found that 35% hydrogen peroxide bleaching gel associated with light activation presented transenamel and transdentinal cytotoxic effects characterized by direct damage to odontoblasts and a decrease of their metabolic activity. For these authors, from a biological point of view, the application of light or heat on the bleaching gel to catalyze HP degradation and speed up tooth bleaching is questionable.

The chemical activation tested increased the diffusion time for the less concentrated gel (20% hydrogen peroxide). It is possible that the faster the diffusion of hydrogen peroxide through enamel and dentin, the greater the amount of peroxide present in the pulp is when considering the same period of time.

When manganese gluconate is added to the bleaching gel, the metal's salts decompose hydrogen peroxide and form free radicals that react with the tooth structure [10]. This decomposition can be seen when the gel is mixed and a considerable amount of oxygen bubbles is formed. Therefore, less hydrogen peroxide molecules were able to reach the artificial pulpal chamber. However, only for the 20% concentration, the chemical activation produced a significant increase in the diffusion time (Table 1). For the 35% concentration there is a greater availability of hydrogen peroxide, so it is likely that the amount of available manganese gluconate was not able to influence the rate of diffusion of peroxide. In a previous study, when manganese gluconate was added to the bleaching gel, a lower concentration of hydrogen peroxide was observed in simulated pulps, but the whitening efficacy was improved [10].

Due to the difficulties in obtaining human teeth with the ideal characteristics for the experiment, bovine incisors were used, providing discs with standardized enamel/dentin thickness. The chemical and physical properties of bovine substrate, such as composition, density, and microhardness, are very similar to human enamel [25]. Bovine and human substrates are also found to have a similar behavior regarding staining and bleaching effects [26].

Furthermore, bovine dentin allows better standardization of permeability conditions, considering the great variability of human dentin permeability characteristics, since the hydraulic conductance and diffusional water flux were shown to be similar between human and bovine dentin [27]. Thus, bovine dentin near cementoenamel junction is considered a suitable alternative for coronal human dentin for *in vitro* studies investigating transdentinal permeability [27].

The spectrophotometric method was previously used to measure the concentration of hydrogen peroxide into the pulp chamber [10, 28, 29]. In this study, this method was used not to measure the concentration but to show the exact moment that the hydrogen peroxide reached the simulated pulp chamber.

The raise of diffusion time can probably diminish the negative effects of the bleaching procedure on pulpal tissue. It is important to take into account that *in vitro* studies may have limited value to simulate clinical conditions. In the vital pulp, the pulpal fluid pressure is capable of reducing inward diffusion of chemicals [30]. In addition, there are sufficient mechanisms in the pulp that protect the tissue from free radicals generated from the reaction of hydrogen peroxide, so that the available levels of hydrogen peroxide would be significantly reduced [31]. Further studies must be conducted to determine the influence of the diffusion time on the amount of peroxide that actually reaches the pulp, as well as its relevance for cytotoxicity on pulpal tissue.

## 5. Conclusion

Considering the limitations of this *in vitro* study, it can be concluded that the 35% hydrogen peroxide diffused more quickly into the pulp chamber than the 20% hydrogen peroxide bleaching gel and that the chemical activation of 20% hydrogen peroxide with manganese gluconate increased the diffusion time of the gel through enamel and dentin.

## Figures and Tables

**Table 1:** Mean (standard of deviation) time (min) for hydrogen peroxide to reach the pulp chamber.

Groups*	No activation	Light activation	Chemical activation
35% HP	87.80 ± 26.23^c^	87.60 ± 23.26^c^	91.80 ± 28.46^c^
20% HP	121.00 ± 37.45^a,b^	97.05 ± 26.81^b,c^	139.90 ± 37.56^a^

*Sets followed by the same letter do not show significant differences (*P* < 0.05).

**Table 2:** Results of Tukey's test for the hydrogen peroxide concentration factor.

Concentration	Mean	SD	Homogeneous sets*	
35% HP	89.07	25.70	A	
20% HP	119.32	38.08		B

*Different letters imply significant differences (*P* < 0.05).

**Table 3:** Results of Tukey's test for the activation factor.

Activation	Mean	SD	Homogeneous sets*	
Light activation	92.33	25.24	A	
No activation	104.40	36.07	A	B
Chemical activation	115.85	40.93		B

*Different letters imply significant differences (*P* < 0.05).

## References

[1] McEvoy SA (1989). Chemical agents for removing intrinsic stains from vital teeth. II. Current techniques and their clinical application. *Quintessence International*.

[2] Dahl JE, Pallesen U (2003). Tooth bleaching—a critical review of the biological aspects. *Critical Reviews in Oral Biology and Medicine*.

[3] Benetti AR, Valera MC, Mancini MN, Miranda CB, Balducci I (2004). In vitro penetration of bleaching agents into the pulp chamber. *International Endodontic Journal*.

[4] Camargo SE, Valera MC, Camargo CH, Gasparoto Mancini MN, Menezes MM (2007). Penetration of 38% hydrogen peroxide into the pulp chamber in bovine and human yeeth submitted to office bleach technique. *Journal of Endodontics*.

[5] Walsh LJ (2000). Safety issues relating to the use of hydrogen peroxide in dentistry. *Australian Dental Journal*.

[6] Eldeniz AU, Usumez A, Usumez S, Ozturk N (2005). Pulpal temperature rise during light-activated bleaching. *Journal of Biomedical Materials Research B*.

[7] Goldstein CE, Goldstein RE, Feinman RA, Garber DA (1989). Bleaching vital teeth: state of the art. *Quintessence International*.

[8] Trindade FZ, Ribeiro AP, Sacono NT (2009). Trans-enamel and trans-dentinal cytotoxic effects of a 35% H_2_O_2_ bleaching gel on cultured odontoblast cell lines after consecutive applications. *International Endodontic Journal*.

[9] Dias Ribeiro AP, Sacono NT, Lessa FCR (2009). Cytotoxic effect of a 35% hydrogen peroxide bleaching gel on odontoblast-like MDPC-23 cells. *Oral Surgery, Oral Medicine, Oral Pathology, Oral Radiology and Endodontology*.

[10] Torres CR, Wiegand A, Sener B, Attin T (2010). Influence of chemical activation of a 35% hydrogen peroxide bleaching gel on its penetration and efficacy—in vitro study. *Journal of Dentistry*.

[11] Batista GR, Barcellos DC, Torres CR, Goto EH, Pucci CR, Borges AB (2011). The influence of chemical activation on tooth bleaching using 10% carbamide peroxide. *Operative Dentistry*.

[12] Travassos AC, Torres CRG, Borges AB, Barcellos DC (2010). In vitro assessment of chemical activation efficiency during in-office dental bleaching. *Operative Dentistry*.

[13] Bauminger BB (1974). Micro method for manual analysis of true glucose in plasma without deproteinization. *Journal of Clinical Pathology*.

[14] Hannig C, Zech R, Henze E, Dreier S, Attin T (2005). Peroxide release into saliva from five different home bleaching systems in vivo. *American Journal of Dentistry*.

[15] Hannig C, Zech R, Henze E, Dorr-Tolui R, Attin T (2003). Determination of peroxides in saliva—kinetics of peroxide release into saliva during home-bleaching with Whitestrips and Vivastyle. *Archives of Oral Biology*.

[16] Hannig C, Willenbücher S, Becker K, Mahony C, Attin T (2006). Recovery of peroxides in saliva during home bleaching—influence of smoking. *Journal of Oral Rehabilitation*.

[17] Hardman PK, Moore DL, Petteway GH (1985). Stability of hydrogen peroxide as a bleaching agent. *General Dentistry*.

[18] Ubaldini AL, Baesso ML, Medina Neto A, Sato F, Bento AC, Pascotto RC (2013). Hydrogen peroxide diffusion dynamics in dental tissues. *Journal of Dental Research*.

[19] Ward M, Felix H (2012). A clinical evaluation comparing two H_2_O_2_ concentrations used with a light-assisted chairside tooth whitening system. *Compendium of Continuing Education in Dentistry*.

[20] Gallagher A, Maggio B, Bowman J, Borden L, Mason S, Felix H (2002). Clinical study to compare two in-office (chairside) whitening systems. *Journal of Clinical Dentistry*.

[21] Jeffery GH, Bassett J, Mendham J, Denney RC (1989). *Voguel’s-Text Book of Quantitative Chemical Analysis*.

[22] Hein DK, Ploeger BJ, Hartup JK, Wagstaff RS, Palmer TM, Hansen LD (2003). In-office vital tooth bleaching—what do lights add?. *Compendium of Continuing Education in Dentistry A*.

[23] Tavares M, Stultz J, Newman M (2003). Light augments tooth whitening with peroxide. *Journal of the American Dental Association*.

[24] Wiegand A, Drebenstedt S, Roos M, Magalhães AC, Attin T (2008). 12-Month color stability of enamel, dentine, and enamel—dentine samples after bleaching. *Clinical Oral Investigations*.

[25] Esser M, Tinschert J, Marx R (1998). Material characteristics of the hard tissues of bovine versus human teeth (in German). *Deutsche Zahnärztliche Zeitschrift*.

[26] Attia ML, Aguiar FH, Mathias P, Ambrosano GM, Fontes CM, Liporoni PC (2009). The effect of coffee solution on tooth color during home bleaching applications. *American Journal of Dentistry*.

[27] Schmalz G, Hiller KA, Nunez LJ, Stoll J, Weis K (2001). Permeability characteristics of bovine and human dentin under different pretreatment conditions. *Journal of Endodontics*.

[28] Berger SB, Tabchoury CP, Ambrosano GM, Giannini M (2013). Hydrogen peroxide penetration into the pulp chamber and dental permeability after bleaching. *General Dentistry*.

[29] Bharti R, Wadhwani K (2013). Spectrophotometric evaluation of peroxide penetration into the pulp chamber from whitening strips and gel: an in vitro study. *Journal of Conservative Dentistry*.

[30] Matthews B, Vongsavan N (1994). Interactions between neural and hydrodynamic mechanisms in dentine and pulp. *Archives of Oral Biology*.

[31] Tse CS, Lynch E, Blake DR, Williams DM (1991). Is home tooth bleaching gel cytotoxic?. *Journal of Esthetic Dentistry*.

